# Local dynamic stability during gait for predicting falls in elderly people: A one-year prospective study

**DOI:** 10.1371/journal.pone.0197091

**Published:** 2018-05-10

**Authors:** Lucia Bizovska, Zdenek Svoboda, Miroslav Janura, Maria Cristina Bisi, Nicolas Vuillerme

**Affiliations:** 1 Department of Natural Sciences in Kinanthropology, Faculty of Physical Culture, Palacky University Olomouc, Olomouc, Czech Republic; 2 Department of Electrical, Electronic and Information Engineering “Guglielmo Marconi”, University of Bologna, Bologna, Italy; 3 EA AGEIS, Universite Grenoble-Alpes, La Tronche, France; 4 Institut Universitaire de France, Paris, France; The Ohio State University, UNITED STATES

## Abstract

Computing the local dynamic stability using accelerometer data from inertial sensors has recently been proposed as a gait measure which may be able to identify elderly people at fall risk. However, the assumptions supporting this potential were concluded as most studies implement a retrospective fall history observation. The aim of this study was to evaluate the potential of local dynamic stability for fall risk prediction in a cohort of subjects over the age of 60 years using a prospective fall occurrence observation. A total of 131 elderly subjects voluntarily participated in this study. The baseline measurement included gait stability assessment using inertial sensors and clinical examination by Tinetti Balance Assessment Tool. After the baseline measurement, subjects were observed for a period of one year for fall occurrence. Our results demonstrated poor multiple falls predictive ability of trunk local dynamic stability (AUC = 0.673). The predictive ability improved when the local dynamic stability was combined with clinical measures, a combination of trunk medial-lateral local dynamic stability and Tinetti total score being the best predictor (AUC = 0.755). Together, the present findings suggest that the medial-lateral local dynamic stability during gait combined with a clinical score is a potential fall risk assessment measure in the elderly population.

## Introduction

Falls are a leading cause of injuries and injury-related deaths in people over 65 years of age [1]. The risk factors for falls in the elderly can be divided into four main groups–behavioural, socioeconomic, biological, and environmental [2]. Generally, the causes of falls are considered intrinsic (related to the person) or extrinsic (related to the environment) [3]. In 30–50% of falls is the cause of the fall extrinsic [4]. It has been shown that ageing is associated with a decline in balance control [5]. This decline generally results in decreased gait stability and increased variability in movement [6–8].

Falls occur mostly in dynamic conditions [9]. The methods to quantify changes in gait stability and variability may be useful for early identification of people at risk of falls and subsequently prevention of falls and fall-related injuries. Furthermore, spatiotemporal gait variables and their fluctuations over time provide relevant information evidencing significant changes in stride length, double support time, step width and stride time variability in elderly fallers compared to non-fallers [10,11]. However, these variables do not reflect the inner structure of a physiological time series and do not provide information about changes in motor behaviour [12]. Without separately evaluating bad and good variance (variability that does or does not affect the final outcome of the task), an increase in the variability of gait pattern may be seen as either an effective or ineffective method for ensuring gait stability [13].

In recent years it has been proven that nonlinear methods, such as local dynamic stability, may reveal age-related changes in gait pattern [14,15]; they may also retrospectively distinguish elderly fallers from non-fallers [16–18] or fall-prone subjects–toddlers–from healthy adults [19]. Terrier and Reynard [15] reported age-related changes in the medial-lateral (ML) local dynamic stability demonstrated by the short-term Lyapunov exponent (LE) computed from upper trunk acceleration. Their results further showed a decreasing local stability with increasing age. Similar results were reported by Buzzi et al. [14] who computed the LE from the vertical displacement time series of lower limb joints and found higher LE values in elderly subjects. According to their results, the LE may indicate age-related changes in gait control; therefore, LE may also have potential in fall risk prediction. To answer this question, Bisi et al. [19] combined the time series of different directions of linear trunk accelerations to compute and compare the LE in toddlers and young adults. They reported higher LE values in toddlers, verifying the expected decreased local stability in toddler gait. Toebes et al. [16] studied the age-gait relationship in elderly fallers and non-fallers using a retrospective approach. Their results implied that the short-term LE computed from combined trunk linear accelerations and angular velocities had the best association with fall history. As shown above, several devices ranging from optoelectronic devices to inertial sensors can be used for gait assessment. Inertial sensors have several advantages compared to optoelectronic devices (cost, portability) and showed a great potential for gait assessment in the elderly population [20].

Fall history observation is another concern in the fall risk assessment. In most of the studies, a retrospective approach was taken where the subjects were questioned on the number of falls experienced several months before the testing procedure itself [20]. However, there is evidence that recall of the number and circumstances of falls often does not reflect the actual state [21]. Furthermore, it is not clear whether the results reflect the fall risk or the actual state as a consequence of previous falls. Considering the inaccuracy of retrospective assessments of fall history, prospective observation of fall occurrence was recommended [22].

The retrospective approaches for estimating fall history may present bias in the interpretation of the results. According to the results of the aforementioned studies, the LE has great potential to be used in the early identification of people at risk of falls. Therefore, the aim of this study is to investigate the LE derived from trunk acceleration during gait and the potential use of the LE as a fall risk assessment measure using a prospective approach. To the best of our knowledge, there have been no studies based on a prospective observation analysing the LE in a controlled in-lab environment. The working hypothesis is that higher LE values precede future falls and therefore, could be used as fall risk predictors. We expect to find higher distinctive strength when comparing multiple fallers and non-fallers.

## Methods

The participants and methods were the same as in the 6-month prospective studies published earlier by our team [23,24]. The baseline measurement was more complex and included several tests; only specific measurements were included in the present work. A brief description of the methods is below.

### Participants

This study was designed as a one-year prospective study focusing on an elderly population. Participants were recruited from the university for elderly (University of the Third Age, Palacky University Olomouc, Olomouc, Czech Republic) and clubs for elderly in Olomouc, Czech Republic according to the following inclusion and exclusion criteria.

Inclusion criteria

- age 60 years and above
- no known neurological or musculoskeletal problem that may affect gait or balance abilities
- ability to stand and walk without any assistance and assisting device

Exclusion criterion

- any injury or surgery on the musculoskeletal system during the last two years before the baseline measurement

The study was approved by the institutional ethics committee (The Ethics Committee of the Faculty of Physical Culture, Palacky University Olomouc, Olomouc, Czech Republic, no. 24/2014). The participants signed written informed consent before the baseline measurement.

### Baseline measurement

During the baseline measurement, the participants filled in the anamnestic questionnaire focusing on their physical condition and fall history in 3 months prior the measurement. If a participant reported any falls, the details were asked. The participants were also examined clinically by the Tinetti Balance Assessment Tool (TBAT) [25], and their gait stability was assessed during 5 minutes of indoor walking (over ground) in a 30 metre long well-lit corridor. Three 3D accelerometers (sampling rate 296.3 Hz, Trigno wireless system, Delsys Inc., Natick, MA, USA) were attached on the trunk near the L5 vertebra and on both shanks approximately 15 cm above the malleolus; acceleration was recorded in the anterior-posterior (AP), vertical (V) and medial-lateral (ML) directions. The sensors on the shanks were added to capture the interaction between the body and the surface (end-point variability). A twenty-five metre long corridor was marked on the floor. The participants were instructed to walk straight in a stable comfortable pace, turn after crossing the mark on the floor and continue to walk at the same comfortable speed. They wore comfortable sport shoes during the test. Data collection started after the first stride of the walking trial to avoid the possible influence of transition to gait on the time series due to a change in speed. The gait speed was computed for each interval from the distance and time (measured by a stop watch) to complete the 25 metre long walking episodes. The average speed was then computed for each participant.

### Fall occurrence observation

After the baseline measurement, the participants were observed for fall occurrence for one year. Every 14 days, the participants received a phone call from one of the research assistants to check if they tripped, slipped or fell. In the event of a trip, slip or fall, the participants included information about their activity during the situation, the exact cause of the situation and the consequences; they were also asked to note the details in the provided notebook. Falls were assessed regularly and categorized in agreement with the recommendation of The Prevention of Falls Network Europe [22]; therefore, a fall was defined as “an unexpected event in which the participants come to rest on the ground, floor, or lower level”. Only falls that occurred during everyday activities were included in the analysis. Thus, falls related to sports activities (12 falls), caused by greater external force (e.g., subjects being suddenly dragged by dogs, 4 falls) and falls related to impeded visual conditions (e.g., walking in the basement storage and unable to turn on the lights, 3 falls) were excluded.

After one year of observation, the cohort was divided into three groups: non-fallers (N, 0 falls), fallers who experienced one fall (F1), and multiple fallers (F2+, two and more falls). The three groups were implemented to ensure consideration of the recurrent fallers, as definitions of fallers are vastly different [26]. The definition of a faller as a person who experienced at least one fall has been used in the literature [27,28]. However, one fall during a one-year period may be a consequence of a random event and not relevant to the fall risk [29].

### Data analysis

**Fig 1:**
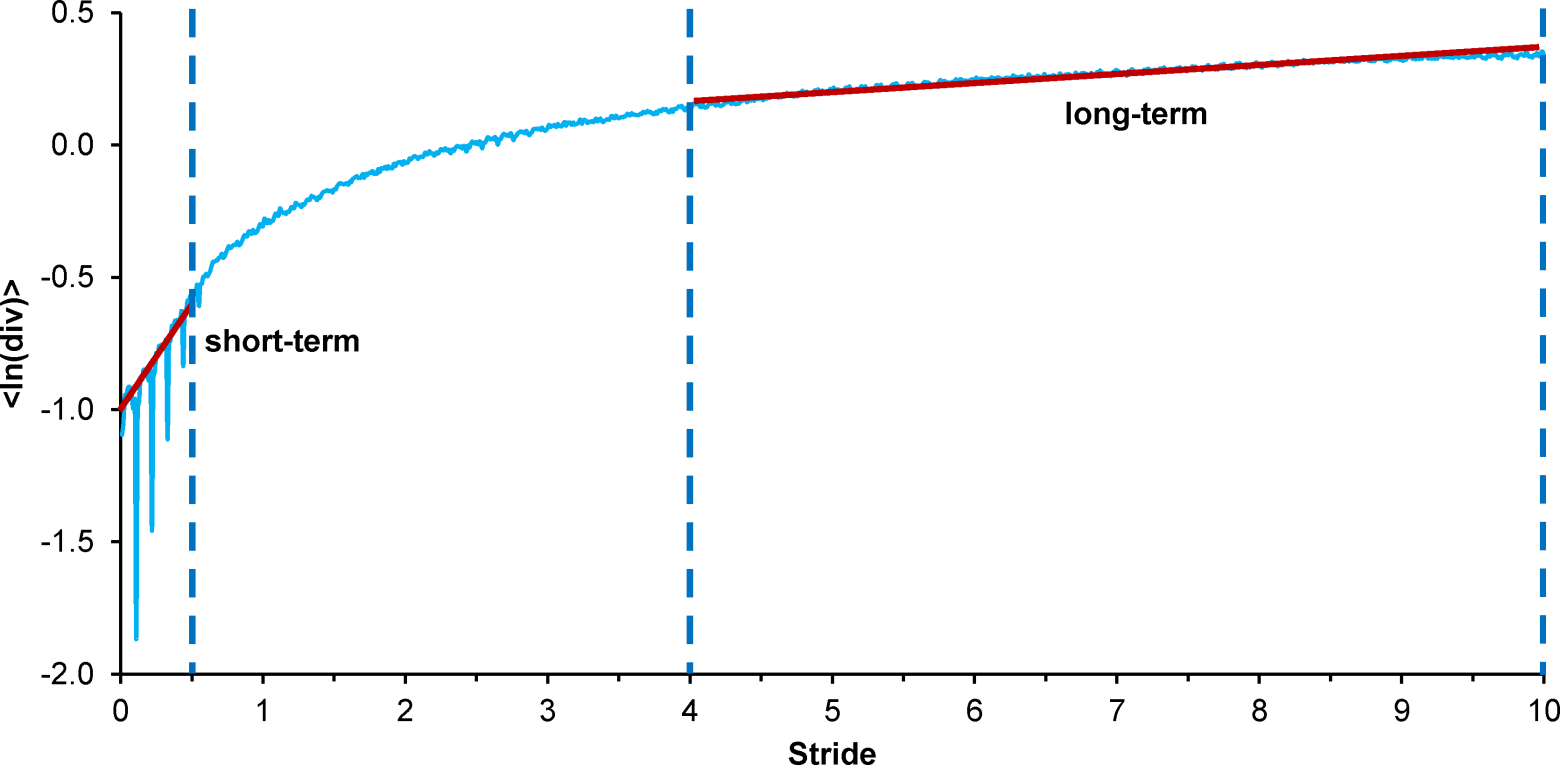
Representation of short- and long-term LE computation. LE are computed as slopes of mean log divergence curve between 0 and 0.5 stride (short-term) and 4 and 10 strides (long-term).

The first 300 data points of recorded data were excluded from the analysis because of the unstable response of the sensors. The last stride before the turn, the U-turn and the first stride after the turn were cut-off from the signal before processing to obtain only the data from straight walking episodes. The cut-off was performed to be sure there was no influence of the turns on the analysed data, thus excluding the gait initiation and termination phases. Riva et al. [30] proved this independence in young healthy adults, but no conclusions were provided for elderly people. The unfiltered signal was then analysed. For the analysis, strides were extracted from the AP trunk acceleration using the heel strike identification as proposed by Zijlstra and Hof [31]. To assess local dynamic stability, short-term and long-term Lyapunov exponents were computed on 150 strides to ensure reliability of indices [32]. For this purpose, the original time series without turns was resampled to 15,000 points to obtain approximately 100 data points per stride. For a state space reconstruction, time delays of 11, 8 and 10 samples for the trunk and 9, 6 and 11 samples for the shanks in the V, ML and AP directions, respectively, were used as a result of the first minimum from the average mutual information function. An embedding dimension of 6 was used as computed by the global false nearest neighbour analysis. To allow comparison between studies, the most widely used algorithm proposed by Rosenstein et al. [33] was used to compute the short-term (over one step, stLE) and long-term LE (over the fourth to tenth stride, ltLE) (Fig 1). Stride frequency was computed from an amplitude spectrum of fast Fourier transformation of the AP trunk acceleration. The computations were performed by a custom Matlab algorithm.

### Statistical analysis

To compare fallers and non-fallers, the Mann-Whitney U test was used since the data did not show a normal distribution in all cases as assessed by the Kolmogorov-Smirnov test. After comparing groups and finding the most significant differences between groups (p < 0.05), a receiver operating characteristic curve analysis (ROC analysis) was used to establish the strength of each significant variable to predict falls in our cohort. Variables were submitted to the ROC analysis separately and combined based on the logistic regression. Specificity and sensitivity were computed for the cut-off points assessed by Youden’s J index (J = max{specificity + sensitivity– 1}). The statistical analysis was performed at a significance level of α = 0.05, however, in each group of variables (clinical scores, short- and long-term LE separately for trunk and shanks) a Bonferroni correction was applied resulting in the adjustment of level of significance to value 0.05/3 = 0.017. Computations were performed with Statistica software (v. 12, StatSoft, Inc., Tulsa, OK, USA) and SPSS Statistics for Windows (v. 18, IBM, New York, NY, USA).

## Results

### General characteristics of participants

A total of 131 elderly people participated in this study (mean age 70.8 ± 6.7 years, height 162.5 ± 7.6 cm, weight 75.3 ± 13.6 kg, body mass index 28.4 ± 4.6 kg.m^-2^). Detailed information about participants and their results for each test are available in the supporting file S1 Data. Based on the prospective fall occurrence observation, participants were divided into three groups as follows: N (81 subjects– 63 females, 18 males), F1 (35 subjects– 31 females, 4 males) and F2+ (15 subjects– 14 females, 1 male). The participants’ characteristics are shown in Table 1. The groups did not differ in age, body mass index nor the number of falls at the baseline (p > 0.05). Significant differences were found between N and F1 in weight (p = 0.037) and height (p = 0.034).

**Table 1:** Demographic and anthropometric characteristics of groups (mean ± standard deviation).

	N (n = 81)	F1 (n = 35)	F2+ (n = 15)	N vs. F1	N vs. F2+	F1 vs. F2+
Age (years)	70.5 ± 6.4	71.4 ± 7.7	71.2 ± 5.3	0.541	0.725	0.919
Height (cm)	163.6 ± 7.8	160.3 ± 7.1	161.5 ± 6.4	0.034	0.335	0.567
Weight (kg)	77.5 ± 14.8	71.6 ± 11.4	72.5 ± 9.3	0.037	0.208	0.789
Body mass index (kg.m^-2^)	28.8 ± 4.6	27.8 ± 4.8	27.8 ± 4.1	0.325	0.459	0.992
Fall history at the baseline–number of falls in group	0.10 ± 0.34	0.20 ± 0.58	0.13 ± 0.35	0.785	0.775	0.975

N–subjects with no fall, F1 –subjects with one fall, F2+–subjects with two and more falls, the last three columns show the p-values for differences between the groups.

### Clinical assessment

The results of the clinical examination are shown in Table 2. The analysis of TBAT scores showed that groups N and F2+ differed in all TBAT scores (balance: p = 0.009; gait: p = 0.015; total: p = 0.000) with lower values for F2+. There was no significant difference between N and F1. Significant differences were found between F1 and F2+ in total score (p = 0.009) with higher values for F1.

**Table 2:** Results of a clinical and basic gait assessment.

	N (n = 81)			F1 (n = 35)			F2+ (n = 15)			p-values		
	median	lower quartile	upper quartile	median	lower quartile	upper quartile	median	lower quartile	upper quartile	N vs. F1	N vs. F2+	F1 vs. F2+
Tinetti score												
balance	16.0	16.0	16.0	16.0	16.0	16.0	16.0	14.5	16.0	0.836	0.009	0.043
Gait	12.0	12.0	12.0	12.0	12.0	12.0	12.0	11.0	12.0	0.433	0.015	0.153
Total	28.0	27.5	28.0	28.0	27.0	28.0	27.0	26.5	28.0	0.850	0.000	0.009
Gait characteristics												
gait speed (m.s^-1^)	1.24	1.16	1.37	1.25	1.13	1.36	1.20	1.10	1.30	0.966	0.204	0.280
stride frequency (Hz)	0.955	0.916	0.987	0.949	0.911	1.020	0.989	0.896	1.006	0.622	0.397	0.949

N–subjects with no fall, F1 –subjects with one fall, F2+–subjects with two and more falls.

### Gait assessment

**Fig 2:**
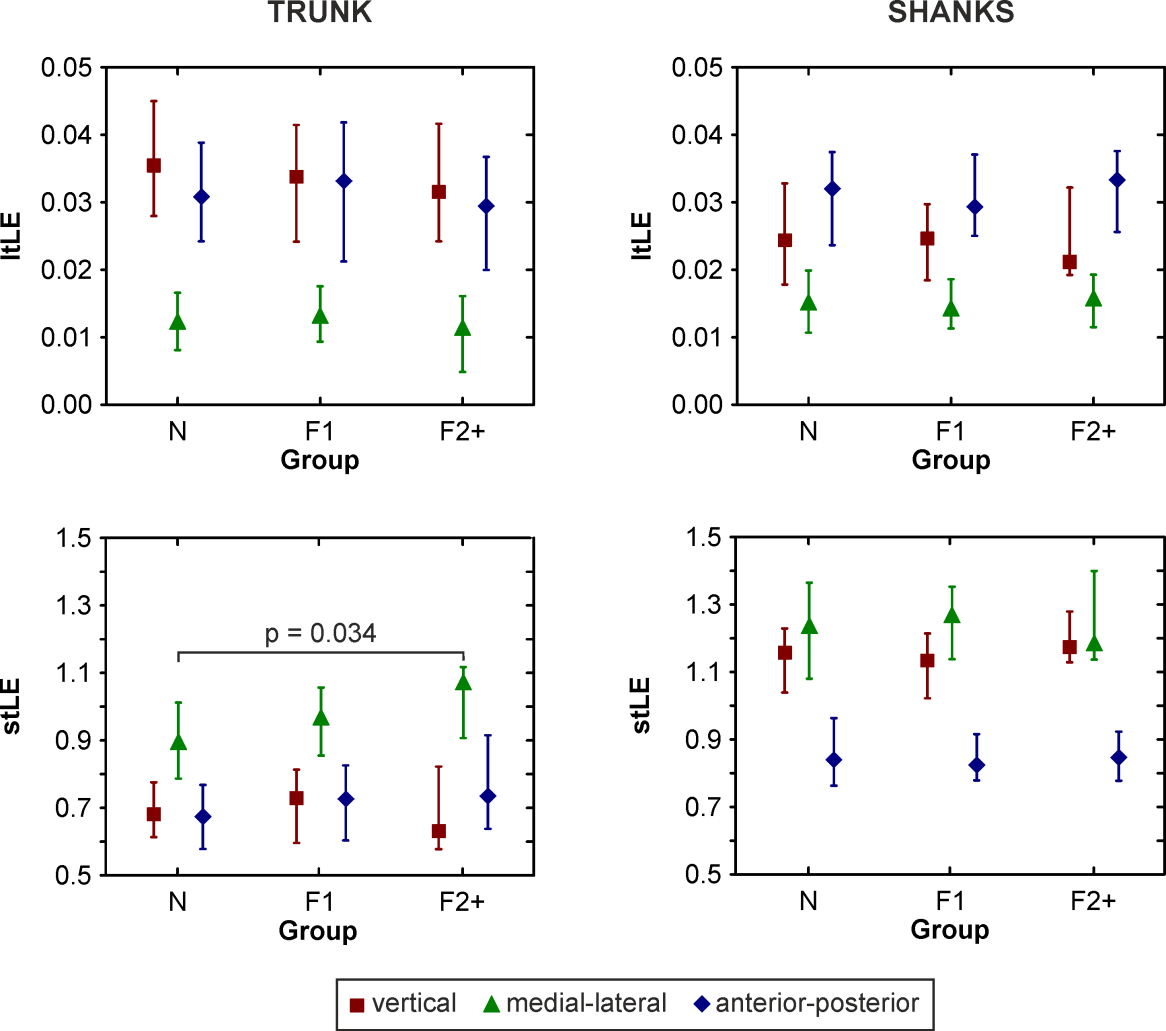
Median group values of long-term (ltLE) and short-term (stLE) Lyapunov exponents for non-fallers (N), fallers experiencing one fall (F1) and multiple fallers (F2+). Error bars indicate lower and upper quartiles.

The gait speed and stride frequency did not differ between any of the groups (p > 0.05) (Table 2). The lowest p-value was found for the trunk ML acceleration in stLE between N and F2+ (p = 0.034) with higher values for F2+ (Fig 2). However, when a Bonferroni correction was applied to the p-value, the difference became insignificant. The N group reached the lowest values of trunk ML stLE, while the F2+ group reached the highest.

### Predictive validity of fall risk assessment measures

In the comparison of N and F2+, the ROC analysis (Table 3) showed the highest area under the ROC curve (AUC) when combining Tinetti balance score, Tinetti total score and trunk ML stLE.

**Table 3:** ROC analysis results for discriminating multiple fallers from non-fallers.

	AUC	Specificity	Sensitivity
Single variable			
Tinetti balance score	0.659	0.89	0.47
Tinetti total score	0.757	0.83	0.67
Trunk stLE ML	0.673	0.85	0.53
Combination of two variables			
Tinetti balance score, Tinetti total score	0.753	0.83	0.67
Tinetti balance score, trunk stLE ML	0.724	0.74	0.73
Tinetti total score, trunk stLE ML	0.755	0.72	0.87
Combination of three variables			
Tinetti balance score, Tinetti total score, trunk stLE ML	0.760	0.72	0.80

AUC–area under the curve, stLE–short-term Lyapunov exponent, ML–medial-lateral.

The individual variables showed AUC values of 0.659–0.757 with Tinetti total score as the best predictor variable. When combining two variables, the AUC increased to the values of 0.724–0.755.

## Discussion

The aim of this study was to assess the potential of local dynamic stability for fall risk prediction in the elderly population. For this purpose, a prospective approach for fall occurrence observation was implemented. The results of the present study showed fair to good strength of Tinetti balance score, Tinetti total score and trunk ML stLE to predict future falls in multiple fallers. The prediction was strengthened when submitting a combination of abovementioned variables to analysis.

The results of the present study showed significant differences between the trunk ML stLE of non-fallers and multiple fallers. The values of trunk stLE in the ML direction increased as the number of observed falls increased, showing a distinct trend of decreased local dynamic stability of the trunk in the ML direction in relation to fall occurrence. This result confirms the previous evidence suggesting that ML movement is crucial for balance control during human gait [15]. The results of the present study showed no significant differences between the gait speed and stride frequency of the groups and no differences in anthropologic data between N and F2+. According to these findings, it may be assumed that the significant difference found in the stLE in the ML direction was not inflicted by differences in gait speed between the groups nor the participants’ individual anthropological characteristics.

There were no significant differences between N and F1 when comparing the clinical test data and gait characteristics, confirming the need to consider at least two falls when defining fallers. As mentioned above, a single fall may be a random event influenced by external factors and not necessarily relevant to actual fall risk [29]. Our study complements the results of Lord et al. [34], who found evidence of similarities between N and F1 in terms of postural stability in women over 65 years of age. In analysing the influence of environment [18], their presented results show that subjects at risk of fall during daily life (F2+) exhibit a decreased ML local stability when walking indoors, which reveals an unexpected decreased ability to overcome small perturbations [35] in a controlled condition.

There were no significant differences in ltLE. The observation of significant differences between N and F2+ only in stLE compared to ltLE is also in agreement with previous studies [16] and is likely related to the progress of an instable situation. The perturbations leading to falls require immediate response so the changes can be accurately observed by stLE [16], which are calculated as a slope of the divergence curve through one step. Compared to ltLE calculated between the 4th and 10th stride, the local stability occurring long after the perturbation likely does not have a strong association with the actual response [16]; therefore, as the results suggest, this local stability is not likely to be crucial for fall risk assessment.

The results of the ROC analysis are not substantial for this cohort. The AUC value of 0.673 when comparing trunk ML stLE between F2+ and N suggests that this variable alone is not suited to distinguish the two groups. This result is not surprising considering the small sample size of F2+; very few participants in the present cohort experienced more than one fall during one year of observation (11.5%). We believe that this result is also related to the results of the clinical examination of the present cohort. Although there were significant differences in the Tinetti scores of fallers and non-fallers (Table 2), the absolute difference was one point at most. The median values for all groups correspond to low risk groups according to the classification provided by Tinetti et al. [25]. The AUC for TBAT balance score was lower compared to AUC of TBAT total score. Furthermore, the AUC of TBAT total score showed higher value compared to trunk ML stLE. The result showing a high TBAT score in fallers is in contrast with other studies investigating fall-related changes using similar procedures–modifications of TBAT [36,37]. There may be several reasons explaining this difference. First, the participants involved in the present study were considerably younger (mean age = 70.8 years, N = 131) than those of Raîche et al. [36] (mean age = 80.0 years, N = 225) and Chiu et al. [37] (mean age of groups 81–83 years, N = 78). The results of the present study are likely related to the inclusion criteria and the recruiting process of the study. Since we wanted to employ non-linear gait characteristics, a high number of gait cycles was needed to obtain reliable results [32]; therefore, one of the inclusion criteria was being able to stand and walk independently. Furthermore, the recruiting process was performed in senior clubs and university which is in contrast to similar studies including participants recruited in hospitals, clinics or through general practitioners [37]. Consequently, we assumed that the participants in the present prospective study were healthy and active elderly people considering their activities such as attending education classes for elderly or socializing in senior clubs.

The results of the present study showed that a combination of clinical examination and gait assessment by local dynamic stability leads to better predictive validity than each test alone. Even though the TBAT total score showed the AUC value comparable to the AUC of combination of two (TBAT total*trunk ML stLE and TBAT total*TBAT balance) or three variables (TBAT total*TBAT balance*trunk ML stLE), the sensitivity increased considerably when using a combination of clinical and gait variables. For future fall prediction, true identification of subjects in risk is crucial. Considering this assumption, the results of this study show that the trunk ML stLE in a combination with TBAT total score has potential for fall risk prediction in high functional elderly subjects generally not considered at fall risk.

There are several limitations present in this study. First of all, the number of multiple fallers is low compared to other groups. Even though a relatively high number of participants with various backgrounds were recruited, we were not able to avoid this consequence. Second limitation is the in-lab setting of the experiment. Future research is needed to compare the predictive ability of variables computed from in-lab and daily-life data collection. Lastly, we used specific analysis for gait assessment, namely local dynamic stability. The results of this analysis depend on the type of the time series used for computation (e.g., angular velocity, acceleration) and the position of the marker or sensor used for data recording [38]. Furthermore, a high number of gait cycles is needed to achieve reliable results [32] making this analysis difficult to perform in clinical settings. Further research focused on other measures and analyses (e.g., orbital stability, recurrence quantification analysis, entropy measures, frequency analysis) is needed to improve the fall risk prediction based on gait analysis.

## Conclusions

The present findings demonstrated that trunk medial-lateral local dynamic stability is a potential marker for fall risk prediction in elderly subjects. The predictive ability improved when combining clinical examination and local dynamic stability. Concerning the clinical results of our cohort, participants in the present study were generally considered at low fall risk. However, the short-term Lyapunov exponents computed from the linear trunk acceleration in the medial-lateral direction displayed a trend of declining local stability with increasing fall occurrence.

## Supporting information

S1 Data Data set. (XLSX)
